# Framework for assessing and easing global COVID-19 travel restrictions

**DOI:** 10.1038/s41598-022-10678-y

**Published:** 2022-04-28

**Authors:** Thien-Minh Le, Louis Raynal, Octavious Talbot, Hali Hambridge, Christopher Drovandi, Antonietta Mira, Kerrie Mengersen, Jukka-Pekka Onnela

**Affiliations:** 1 Department of Biostatistics, Harvard T.H. Chan School of Public Health, Boston, MA USA; 2 Università della Svizzera Italiana, Lugano, Switzerland; 3 School of Mathematical Sciences, Faculty of Science, Queensland University Technology, Brisbane, Australia

**Keywords:** Public health, Statistics, Influenza virus

## Abstract

During the COVID-19 pandemic, many countries implemented international travel restrictions that aimed to contain viral spread while still allowing necessary cross-border travel for social and economic reasons. The relative effectiveness of these approaches for controlling the pandemic has gone largely unstudied. Here we developed a flexible network meta-population model to compare the effectiveness of international travel policies, with a focus on evaluating the benefit of policy coordination. Because country-level epidemiological parameters are unknown, they need to be estimated from data; we accomplished this using approximate Bayesian computation, given the nature of our complex stochastic disease transmission model. Based on simulation and theoretical insights we find that, under our proposed policy, international airline travel may resume up to 58% of the pre-pandemic level with pandemic control comparable to that of a complete shutdown of all airline travel. Our results demonstrate that global coordination is necessary to allow for maximum travel with minimum effect on viral spread.

## Introduction

With more than 129 million confirmed cases and 2.8 million deaths globally as of March 31, 2021^1^, the COVID-19 pandemic has had an enormous impact on the world. The pandemic damaged the global economy, which shrank by 5.2$\%$ in 2020, the largest recession since World War II^2^. With a patchwork of travel bans in place worldwide, the tourism industry has been severely affected, with estimated losses of 900 billion to 1.2 trillion USD and tourism down 58$\%$−78$\%$^3^. The airline industry has also suffered heavily, with 43 airlines declaring bankruptcy and 193 of 740 European airports at risk of closing^4,5^. To contain the pandemic, most countries took a two-pronged approach. First, they attempted to slow the spread of the disease internally by implementing various non-pharmacological interventions, such as social distancing, using face coverings, and closing businesses and schools. Second, they attempted to reduce the number of imported cases by implementing travel restrictions. While travel restrictions benefit the community by preventing importation of some cases, these policies end up costing the global economy an estimated 400 billion USD and millions of jobs each month^6–8^. The gravity of the situation highlights the need for balance between protecting the health of the public and mitigating the short- and long-term economic damage related to infection control efforts.

The effectiveness of travel restrictions has been investigated in many studies^9–15^ (see^16–18^ for systematic reviews). Most of these studies suggest that travel restrictions are primarily effective at the early stage of a pandemic and may help to delay a pandemic up to 4–6 months^11,18^. However, the effect of travel restrictions wanes over time as cases are inevitably imported. Furthermore, the effect of travel restrictions is minimal relative to that of internal mitigation measures such as social distancing and mask wearing. Many researchers have concluded that continued use of travel restrictions is not worth the economic trade-off^6,17^. Although many studies have examined the effectiveness of travel restrictions, limited research has focused on the best way to lift these restrictions while still protecting health^16^. Costantino et al.^19^ and Linka et al.^20^ studied partial removal of travel bans and urged caution for regions opening themselves up to regions with a more dire public health situation. Russell and colleagues went a step further by suggesting scenarios in which a country may want to leave travel restrictions in place^21^. The authors argued that based on existing pandemic data and travel data, policymakers should first reconstruct the pandemic situation in each country and then estimate the number of imported cases they receive from each country. The ratio of imported cases to internal cases, together with the effective reproduction number, should then be used to decide whether travel restrictions are needed in that country. While these studies emphasize the important roles of imported and internal cases, none of them recommend specific strategies for easing travel restrictions or propose ways to coordinate them effectively to minimize health risks.

Our paper aims to address this gap in the literature. We developed a flexible network meta-population model for comparing the effectiveness of international travel policies, with a focus on evaluating the benefit of policy coordination. Because the epidemiological parameters of countries are unknown, they need to be estimated from data, a task usually accomplished using the likelihood function. However, complex stochastic models of infectious disease transmission often do not have computationally tractable likelihood functions. To overcome this limitation, we relied on a class of likelihood-free methods called approximate Bayesian computation (ABC). We then used our framework to examine two hypothetical travel-regulation policies that allow people to move from one country to another. The goal was to ensure that a country’s public health situation does not deteriorate after the country adopts the proposed travel policy. Theoretical results are provided to support the two proposed approaches. We also used simulation to compare the effectiveness of our recommended policies with existing travel restriction policies, such as a 14-day quarantine for all arrivals and a 14-day quarantine only for people returning from high-risk countries. Simulations indicate that our proposed travel policies would allow for more incoming travelers while maintaining control of the pandemic. Finally, we discuss how our proposed policies can be implemented in practice.

## Results

### Simulation studies

#### Effectiveness of travel policies

We determined the effectiveness of six different travel regulation policies labeled P1 through P6 for four synthetic (simulated) countries. In these simulation settings, we considered only four representative countries, where each country has a basic reproduction number $R_0$ in the following ranges: 0.47–0.9, 0.9–1, 1–1.1, and 1.1–6.47. Here $R_0= \alpha /(\gamma + \beta )$ and the range of $R_0$ values is motivated by the study of Rahman et al. (2020)^33,46^ (see the Methods for the model parameters). Policy effectiveness is compared in terms of the percentage of people allowed to travel relative to the pre-pandemic period and the pandemic situation in the country if it adopted a given policy. The first two policies were the most extreme: all countries are fully open or fully closed, denoted as policies P1 and P2, respectively. We investigated the effectiveness of the four remaining policies by having a country adopt the given policy while all other countries remain fully open. Under P3, the receiving country requires a 14-day quarantine for all arrivals. Under P4, the country requires 14-day quarantine only for travelers from high-risk countries. A country is considered high risk if the average number of active confirmed daily cases exceeds 20 per 100,000 people in the last 2 weeks^22,36^. Under P5, the receiving country adopts the simplified version of the proposed average control policy, where travel is regulated such that the average number of daily undetected infected cases is at most 10$\%$ higher than the maximum number of daily cases under P2. In P6, the country adopts the simplified version of the proposed probability control policy, but travel is regulated such that the average number of daily undetected infected cases is at most 10$\%$ higher than the maximum daily cases under P2 with a probability of at least 90$\%$. Detailed simulation settings and comprehensive outputs for the effectiveness of different policies can be found in the Supplementary Materials.

**Table 1:** Results for effectiveness of travel regulation policies P1 through P6 for synthetic data. Shown are 2.5th and 97.5th percentiles of travel and health outcomes for the policies using estimated epidemiological parameters to simulate epidemic and travel data. G1, G2, and G3 denote countries in Group 1, 2, and 3, respectively. The relative change in the number of cases (including detected and undetected), RU, is the difference at the end and at the beginning of the regulated period divided by the number of cases at the beginning of the period; The relative change in the number of confirmed cases, RA, is the difference in the number of confirmed cases at the end and at the beginning of the regulated period divided by the number of confirmed cases at the beginning of the period; IA is the percentage of incoming travelers who will eventually move to the active confirmed category after arrival; Tc is the percentage of inbound travel capacity; and Te is the percentage of expected of inbound travel.

		P1	P2	P3	P4	P5	P6
G1	RU	(2.53, 3.20)	(0.06, 0.27)	(0.64, 0.92)	(0.88, 1.26)	(0.06, 0.27)	(0.06, 0.26)
	RA	(1.58, 2.14)	(0.08, 0.27)	(0.86, 1.15)	(0.99, 1.36)	(0.08, 0.27)	(0.08, 0.27)
	IA	(0.09, 0.11)	(0.00, 0.00)	(0.09, 0.11)	(0.09, 0.11)	(0.00, 0.00)	(0.00, 0.00)
	Tc	100%	0%	100%	100%	34%	0%
	Te	100%	0%	5%	89%	34%	0%
G2	RU	(1.50, 2.05)	(0.45, 0.84)	(0.63, 1.02)	(0.86, 1.32)	(0.46, 0.84)	(0.45, 0.84)
	RA	(0.99, 1.37)	(0.37, 0.64)	(0.60, 0.90)	(0.71, 1.04)	(0.37, 0.64)	(0.36, 0.64)
	IA	(0.09, 0.11)	(0.00, 0.00)	(0.09, 0.11)	(0.09, 0.11)	(0.00, 0.00)	(0.00, 0.00)
	Tc	100$\%$	0$\%$	100$\%$	100$\%$	60$\%$	0$\%$
	Te	100$\%$	0$\%$	5$\%$	89$\%$	60$\%$	0$\%$
G3	RU	(6.28, 6.65)	(6.30, 6.67)	(6.28, 6.65)	(6.28, 6.65)	(6.28, 6.65)	(6.28, 6.65)
	RA	(5.32, 5.56)	(5.33, 5.57)	(5.32, 5.56)	(5.32, 5.56)	(5.32, 5.56)	(5.32, 5.56)
	IA	(0.00, 0.00)	(0.00, 0.00)	(0.0, 0.00)	(0.00, 0.00)	(0.0, 0.00)	(0.00, 0.00)
	Tc	100$\%$	0$\%$	100$\%$	100$\%$	34$\%$	0$\%$
	Te	100$\%$	0$\%$	5$\%$	100$\%$	34$\%$	0$\%$

Table 1 demonstrates how the different policies affect travel and each receiving country’s pandemic situation. Overall, our proposed average control policy, P5, performed best at balancing the number of travelers and health outcomes. To give an informative assessment of the effectiveness of travel restrictions on the pandemic, we report the outputs by stratifying the countries into three groups: Group 1 (G1) consists of countries with the effective reproduction number $R_t$ less than 0.9, Group 2 (G2) consists of countries with $R_t$ between 0.9 and 1.1, and Group 3 (G3) consists of countries with $R_t$ greater than 1.1, where $R_t = \{\alpha S(t)\}/\{(\gamma + \beta )P\}$ and *P* is the size of the population^46^. For all groups, the number of expected inbound travelers was highest for P1, followed by P4, then P5. To satisfy the requirements dictated by P6, countries had to eliminate inbound travel, rendering this policy equivalent to P2. Under P3 and P4, approximately $0.09\%$−$0.11\%$ of inbound travelers of Group 1 and Group 2 would become active confirmed. Therefore, if a receiving country has limited healthcare resources, it may experience challenges adopting P3 or P4. In terms of health outcomes, as expected, the more stringent the travel restrictions, the smaller the number of cases. We also observed that travel restrictions were very effective for countries in Groups 1 and 2, with clear distinctions in the country’s pandemic situation upon adoption of the different travel regulation policies. The changes in cases and confirmed cases for countries in Group 3 were quite similar regardless of whether these countries fully closed or fully open their borders. Countries in Group 3 have the least to gain from travel restrictions since cases in these countries will continue to climb whether the borders are open or closed. Even if a country in this group were to open its border, the imported cases would still constitute only a small proportion of the overall (internal and imported) cases. The epidemic in these countries is most effectively controlled by extensive vaccination of the population as well as large-scale adoption of non-pharmaceutical interventions (primarily masking and social distancing).

#### Effectiveness of travel policy coordination

To study the effectiveness of policy coordination, we investigated the percentage of people allowed to travel and the overall worldwide pandemic situation under different globally coordinated travel policy scenarios labeled S1 through S6. Here we considered eight synthetic (simulated) countries, where countries 1 and 2 have $R_0$ between 0.47 and 0.9, countries 3 and 4 have $R_0$ between 0.9 and 1, countries 5 and 6 have $R_0$ between 1 and 1.1, and countries 7 and 8 have $R_0$ between 1.1 and 6.47. The first two scenarios are the most extreme, where all countries are fully open or fully closed, denoted by S1 and S2, respectively. We used S3 to denote the scenario where all countries require a 14-day quarantine for all arrivals; S4 to denote the scenario where all countries use the simplified version of the average control policy; S5 to denote the scenario where countries 1, 3, 5, and 7 require a 14-day quarantine for all arrivals while countries 2, 4, 6, and 8 are fully closed to inbound travel; and S6 to denote the scenario where countries 1, 3, 5, and 7 use the simplified version of the proposed average control policy while countries 2, 4, 6, and 8 are fully closed to inbound travel. We used the same outcome measurements as in the previous simulation. Finally, we also evaluated the global coordination effectiveness by averaging the above measurements for all countries.

**Table 2:** Results for effectiveness of travel policy coordination in scenarios S1 through S6 for synthetic data. Shown are 2.5th and 97.5th percentiles of travel effects and health outcomes for scenarios S1 through S6 using estimated epidemiological parameters to simulate epidemic and travel data. G denotes all countries. See Table 1 caption for more information.

		S1	S2	S3	S4	S5	S6
G	RU	(10.68, 11.56)	(2.65, 3.06)	(4.02, 4.51)	(2.66, 3.07)	(3.45, 3.92)	(2.66, 3.07)
	RA	(8.13, 8.89)	(2.77, 3.13)	(4.92, 5.43)	(2.77, 3.14)	(4.08, 4.55)	(2.77, 3.13)
	IA	(1.57, 1.68)	(0.00, 0.00)	(1.57, 1.68)	(0.00, 0.01)	(0.80, 0.85)	(0.00, 0.00)
	Tc	100$\%$	0$\%$	100$\%$	50$\%$	50$\%$	25$\%$
	Te	100$\%$	0$\%$	5$\%$	50$\%$	3$\%$	25$\%$
G1	RU	(11.16, 12.23)	(0.59, 0.93)	(3.17, 3.61)	(0.60, 0.94)	(1.84, 2.22)	(0.59, 0.93)
	RA	(9.01, 9.95)	(0.74, 1.06)	(4.42, 4.90)	(0.75, 1.08)	(2.52, 2.91)	(0.75, 1.07)
	IA	(1.98, 2.09)	(0.00, 0.00)	(1.98, 2.10)	(0.00, 0.01)	(1.00, 1.04)	(0.00, 0,00)
	Tc	100$\%$	0$\%$	100$\%$	64$\%$	50$\%$	32$\%$
	Te	100$\%$	0$\%$	5$\%$	64$\%$	3$\%$	32$\%$
G2	RU	(12.13, 13.29)	(1.54, 2.13)	(2.98, 3.68)	(1.54, 2.13)	(2.50, 3.19)	(1.54, 2.13)
	RA	(8.14, 9.14)	(1.62, 2.13)	(4.08, 4.81)	(1.62, 2.13)	(3.33, 4.04)	(1.62, 2.13)
	IA	(1.77, 1.89)	(0.00, 0.00)	(1.77, 1.89)	(0.00, 0.01)	(0.89, 0.95)	(0.00, 0.00)
	Tc	100$\%$	0$\%$	100$\%$	64$\%$	50$\%$	32$\%$
	Te	100$\%$	0$\%$	5$\%$	64$\%$	3$\%$	32$\%$
G3	RU	(7.31, 7.45)	(6.94, 7.08)	(6.94, 7.08)	(6.94, 7.08)	(6.95, 7.08)	(6.94, 7.08)
	RA	(7.25, 7.35)	(7.10, 7.20)	(7.12, 7.22)	(7.10, 7.20)	(7.11, 7.21)	(7.10, 7.20)
	IA	(0.77, 0.82)	(0.00, 0.00)	(0.77, 0.82)	(0.00, 0.00)	(0.42, 0.45)	(0.00, 0.00)
	Tc	100$\%$	0$\%$	100$\%$	7$\%$	50$\%$	3$\%$
	Te	100$\%$	0$\%$	5$\%$	7$\%$	3$\%$	3$\%$

Table 2 reports the effectiveness of different coordinated responses in the three groups of countries along with a global average. Under S4, where all countries use the proposed average control policy, the expected inbound travel increased up to $50\%$ of normal travel, and the global pandemic situation was similar to that seen in scenarios where the borders are closed. These findings demonstrate that a global response is critical for containing the pandemic while maximizing safe travel.

### Real data analysis

We used pandemic data from the Johns Hopkins University coronavirus data repository through May 31, 2020^25^. Flight data are from the Official Airline Guide (OAG). Because only data for January and February 2020 are available from OAG, we estimated flight data for other time periods using the OpenSky Network database^26–28^. This database tracks the number of flights from one region to another over time, which can be used to calculate the rate of flight reduction and to estimate flight data for other months. We considered the starting day for each country to be the first day the country exceeded 500 total confirmed cases, because the estimation for the number of undetected infectious people is unstable during each country’s early pandemic period. For similar reasons, we only analyzed the 92 countries whose total number of confirmed infected cases exceeded 500 by April 15, 2020. For the modeling purpose, the remaining countries were combined into a single fictional country labeled “Other.” Here, “Other” represents an amalgamation of countries with very few cases. We assume that most outbound travelers from this country are susceptible; we randomly assigned them a combination of parameters consistent with a small value of $R_0$ when running the global model. The inbound and outbound travel flows of the underlying small countries are combined in a single daily inbound and outbound flow.

To demonstrate the ability of our model to capture the real evolution of the pandemic, we fit it to real data as follows. The fitting period starts when a country exceeds 500 total confirmed cases for the first time before May 31, 2020. The transmission rate $\alpha$ may change during the study. Here, we allow two different values for $\alpha$ for each country, denoted by $\alpha _1$ and $\alpha _2$; both need to be estimated from data and the location of the country specific change point also needs to be estimated from data.

**Figure 1:**
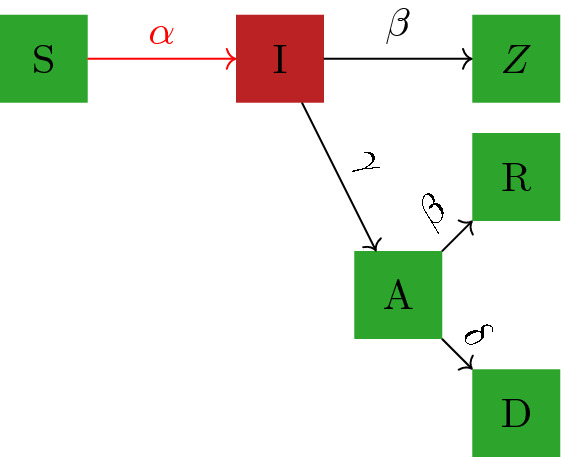
Schematic of the local epidemiological compartmental model which describes the state of the country at any given time. The population of each country is divided into six mutually exclusive compartments: susceptible (*S*), undetected infected (*I*), active confirmed (*A*), confirmed recovered (*R*), confirmed deceased (*D*), and unconfirmed removed (*Z*). The basic reproductive number in this model is given by $R_0= \alpha /(\gamma + \beta )$.

**Figure 2:**
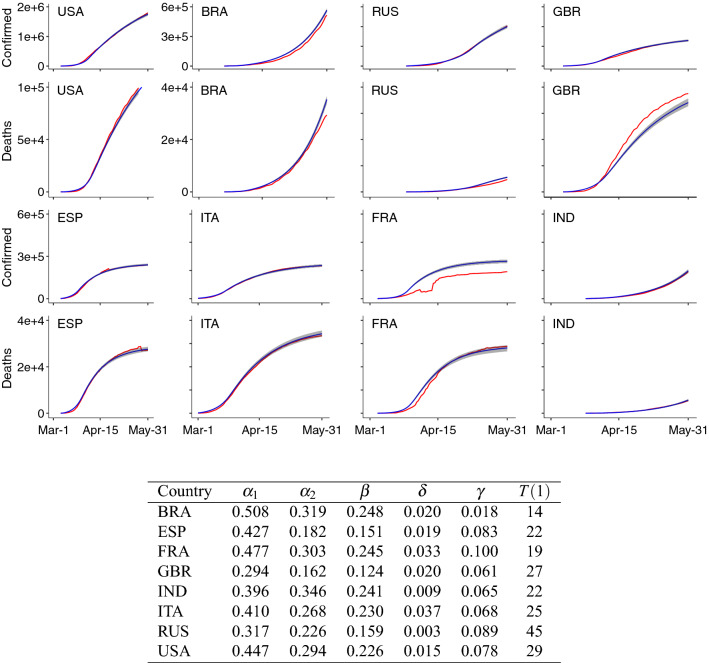
Model fit for different countries. For each country, the fit is demonstrated by the number of accumulated confirmed cases and the accumulated confirmed deaths. In each plot, the red line is the real data, the blue line is the median fitted values, and the shaded region is the 95$\%$ confidence interval. Eight countries are fitted including: the United States of America (USA), Brazil (BRA), Russia (RUS), the United Kingdom (GBR), Spain (ESP), Italy (ITA), France (FRA), and India (IND). Estimated parameter values for the eight countries are as below. Here *T*(1) is the change point of the transmission rate $\alpha$, such that $\alpha$ = $\alpha _1$ when $t \le T(1)$ and $\alpha$ = $\alpha _2$ when $t > T(1)$.

Figure 2 shows the fit of our model for eight countries with the highest number of accumulated confirmed cases up to May 31, 2020. Overall, the figure shows that our model effectively captures the real data (red), as the estimated line (blue) is very close to the observed line and is contained within the confidence interval.

To understand how different travel restriction policies affect the pandemic both globally and in each country, we used the estimated parameters for all countries before May 31 together with the travel data to simulate the course of the pandemic during the first 14 days of June 2020 under four different travel regulation scenarios. In the first scenario, where all countries are fully open, we used 2019 travel data from the pre-pandemic period. In the second scenario, we used 2020 travel data during the COVID-19 pandemic. In the third scenario, all countries fully closed their borders. In the fourth scenario, we supposed all countries use the simplified average control policy.

**Figure 3:**
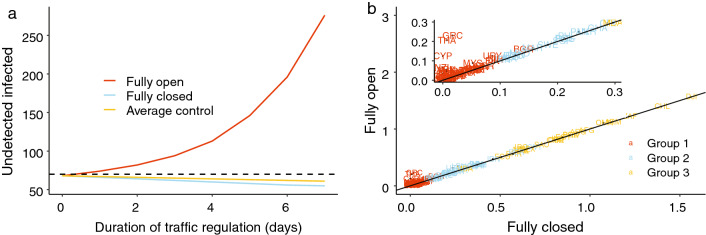
(**a**) Prediction of the average number of undetected infected cases for different travel regulation policies. “Fully open” indicates no travel restrictions are in place, “Fully closed” indicates no travel is permitted, and “Average control” denotes our proposed policy whereby the number of daily undetected infected cases should stay below a threshold of $c=70$ (the dashed line) on average. (**b**) Scatter plot for the relative change in the total number of new cases for each country in the two most extreme scenarios, fully closed and fully open for the first two weeks of June 2020. The 97.5th percentile value of relative change in each country’s number of new cases under the “Fully closed” scenario (x-axis) is plotted versus the corresponding number for the “Fully open” scenario (y-axis). The closer a country is to the reference line $x=y$, the less benefit that country gains from travel restrictions.

Table 3 reports the relative change in the total number of cases, the total number of confirmed cases, and the inbound travel capacity for different countries. We evaluated the global effect of the pandemic for all countries and for the three groups of countries based on their $R_t$ values as defined earlier. The proposed simplified average control policy was the most effective in controlling the pandemic while still maximizing travel capacity. When countries used the proposed policy, the relative change in cases and confirmed cases was similar to those observed under the fully closed scenario. At the same time, the global travel rate remained as high as 58$\%$ compared to the fully open scenario. Additionally, the countries belonging to Group 1 benefited the most from travel restrictions with very little change in cases, even when comparing the most extreme scenarios. The 95$\%$ confidence interval for the relative change in cases for Group 1 was between 0.02 to 0.03 under the fully closed scenario and between 0.05 to 0.06 under the fully open scenario. Group 2 countries also saw only nominal benefit when closing the border compared to the fully open case. The 95$\%$ confidence interval for the relative change in cases decreased to (0.22, 0.26) under the fully closed scenario and to (0.24, 0.27) under the fully open scenario. Countries in Group 3 benefited the least from travel restrictions. The relative change in cases was between 0.80 to 0.84 under the fully closed scenario and between 0.81 to 0.85 under the fully open scenario. Figure 3b demonstrates how much different countries benefited from travel restrictions during the first 2 weeks of June. Greece (GRC), Thailand (THA), Cyprus (CYP), and New Zealand (NZL) benefited most from border closure.

**Figure 4:**
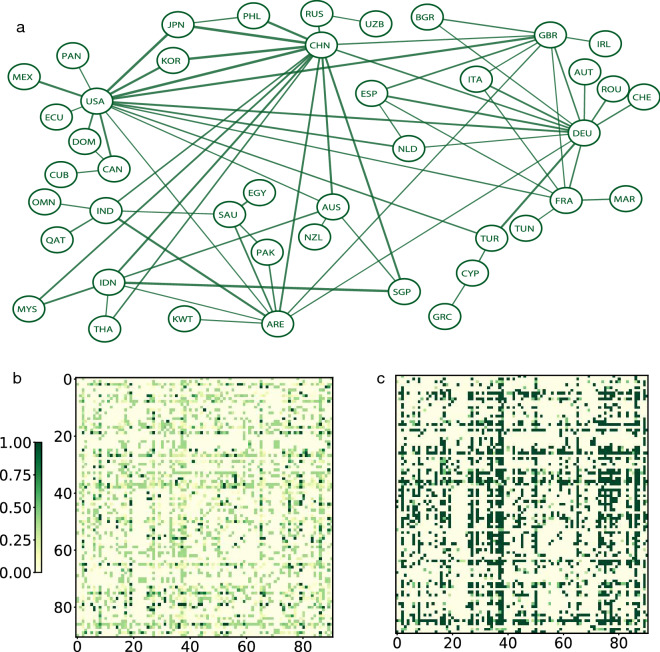
**(a)** Airline traffic network of countries in the top 5$\%$ of mutual global airline travel volume in the first two weeks of June 2020. Each node corresponds to a country and thicker edges carry more travel. **(b)** Heat map of empirical travel volume in the first two weeks of June 2020 (compared to the first two weeks of June 2019), and **(c)** heat map of hypothetical travel volume in the first two weeks of June 2020 under our proposed policy (compared to the first two weeks of June 2019).

**Figure 5:**
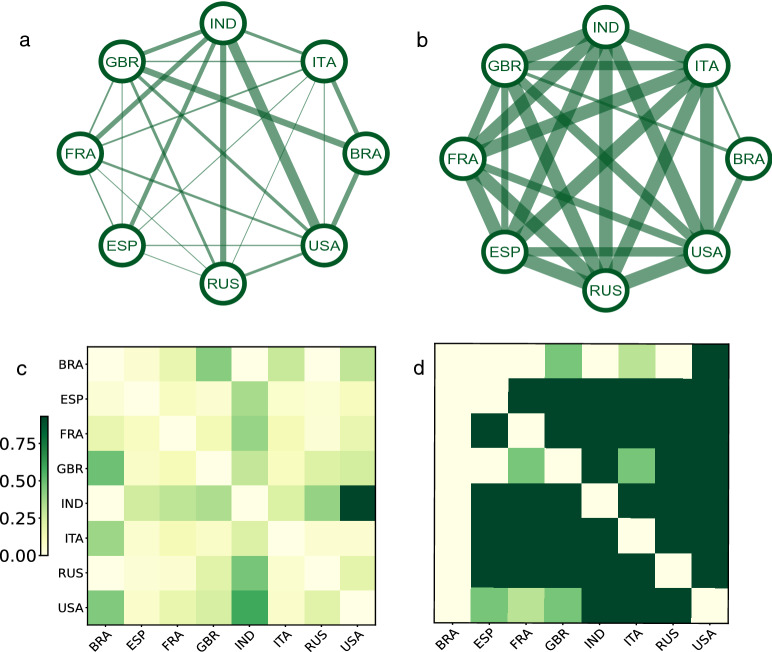
Airline travel network visualizations and corresponding heat maps for eight countries: the United States of America (USA), Brazil (BRA), Russia (RUS), the United Kingdom (GBR), Spain (ESP), Italy (ITA), France (FRA), and India (IND). **(a)** Actual airline travel volume in the first two weeks of June 2020; **(b)** hypothetical travel volume in the same period following our proposed policy; heat maps of **(c)** empirical and **(d)** hypothetical travel volumes normalized by travel volumes from the first two weeks of June 2019.

Finally, we also saw a huge reduction in global travel under the shutdown scenario with 2020 travel amounting to only 33$\%$ of 2019 travel. Figure 4a demonstrates the airline traffic of countries in the top 5$\%$ of mutual global travel volume in the first two weeks of June 2020. Figure 4b, c are travel volume heat maps during the first two weeks of June 2020 and the hypothetical travel volume under our proposed policy compared to the same period of 2019. Figure 4b again shows that under existing travel restriction policies, there is a large reduction in travel in 2020 compared to the same period in 2019. Figure 4c demonstrates that the proposed policy allows significantly higher travel volume compare to existing travel policies. Figure 5 demonstrates the travel volume among the top eight countries with the highest number of accumulated confirmed cases before May 31, 2020.

**Table 3:** Results for effectiveness of travel policy coordination for empirical data. Shown are 2.5th and 97.5th percentiles of relative change in the pandemic situation and percentages of inbound travelers from different groups of countries for different travel regulation scenarios. G denotes all countries; G1, G2, and G3 denotes countries in Group 1, 2, and 3, respectively; RU is the relative change in the number of cases (including detected and undetected), and RA is the relative change in the number of cases that were confirmed.

		2019 data	2020 data	Fully closed	Proposed
G	RU	(0.28, 0.31)	(0.27, 0.30)	(0.26, 0.29)	(0.26, 0.29)
	RA	(0.29, 0.31)	(0.28, 0.30)	(0.27, 0.29)	(0.27, 0.29)
	Inbound travel	100$\%$	33$\%$	0$\%$	58$\%$
G1	RU	(0.05, 0.06)	(0.03, 0.04)	(0.02, 0.03)	(0.02, 0.03)
	RA	(0.04, 0.05)	(0.03, 0.04)	(0.02, 0.03)	(0.02, 0.03)
	Inbound travel	100$\%$	29$\%$	0$\%$	55$\%$
G2	RU	(0.24, 0.27)	(0.23, 0.26)	(0.22, 0.26)	(0.22, 0.26)
	RA	(0.25, 0.28)	(0.24, 0.27)	(0.24, 0.27)	(0.24, 0.27)
	Inbound travel	100$\%$	37$\%$	0$\%$	66$\%$
G3	RU	(0.81, 0.85)	(0.80, 0.84)	(0.80, 0.84)	(0.79, 0.84)
	RA	(0.81, 0.85)	(0.81, 0.84)	(0.81, 0.84)	(0.80, 0.84)
	Inbound travel	100$\%$	36$\%$	0$\%$	54$\%$

## Discussion

In this paper, we proposed a flexible network meta-population model for comparing the effectiveness of international travel policies and for assessing the benefit of international travel policy coordination. Using a mixture of simulation and theoretical findings, we showed that our proposed average control policies can effectively preserve global public health by reducing the number of cases while allowing international travel, thereby preserving the global economy. Our results show that globally coordinated travel policies are not only necessary for resuming international travel, but that it is also possible to accomplish this goal with minimal effect on public health relative to full border closure. Our proposed framework is robust as new and potentially more transmissible variants of SARS-CoV-2 continue to arise in the coming months and years. The model needs to be re-estimated using new data, but the framework itself remains valid.

On the technical side, we proposed a marginal approach for estimating the epidemiological parameters for each country in a global network meta-population model. This approach helped overcome some of the difficulties of simultaneously or jointly estimating the model parameters.

Our statistical approach has one main limitation: we try to control a hidden state of the model, the number of undetected infected cases *I*(*t*), which by definition is not available in the collected data. Nevertheless, the undocumented infectious cases are an important category for spreading the disease and their numbers need to be estimated regularly^30,31^. Given the model, with the available data, we can approximate the hidden state *I*(*t*) by using the approximation $I(t) \approx \{U(t+1) - U(t)\}/{\gamma }$, where $U(t) = A(t) + R(t) + D(t)$ and $\gamma$ is the identification rate that can be estimated from data. High-quality data is critical for tracking the number of undetected infected cases. In public health settings, one of the best strategies to estimate *I* is regular use of randomized serology testing^31,32,34^.

Our model assumes active confirmed cases do not spread the disease. Since patients with COVID-19 may sometimes spread the disease to healthcare workers or their family members when they are in quarantine, this assumption may not hold in practice. Another limitation is that we used a conservative approach to model the global pandemic by assuming that travelers are either susceptible or undocumented infected. However, in reality, some travelers may be recovered confirmed or recovered unconfirmed cases, and therefore cannot infect anyone after arrival in another country. If this fact is taken into account, the number of people traveling may be higher than the currently reported numbers suggest. Unfortunately, the way empirical real data are currently reported does not reflect this fact.

The proposed travel regulation policies are designed for a meta-population model with local pandemic components as described in Warne et al.^29^. Replacing this local infectious model^29^ with different models such as SEIR or MSEIR as discussed in^35^ can be easily accommodated by our modeling architecture. It is also possible to modify the proposed regulation policies to adapt to new virus variants.

As with any public health crisis, there are many possibilities for mitigating the impact of the pandemic. Beyond the 14-day quarantine for all arrivals policy, many countries currently implement more relaxed travel policies. For example, the United Kingdom, Switzerland, and Portugal divide countries into two main zones, “red” and “green,” and the zone of a country is updated every two weeks. Under this policy, a traveler from a green zone country does not need to quarantine upon arrival whereas different requirements apply to a traveler from a red zone country. The cut-off values currently used for zoning are subjective. For example, the threshold for the red zone is 40/100,000 in the United Kingdom (U.K.), 60/100,000 in Switzerland, and 500/100,000 in Portugal; the numerator in each is the number of positive cases detected in the past 14 days in the country^22,36^. Defining a cut-off solely based on incidence rate can however fail if the pandemic takes a quick turn to the worse. As shown in Table S5, under this policy (P4), about 0.03%−0.04% of infected travelers from a green zone country go undetected; in contrast, this number is essentially 0% when using our average control policy (P5).

**Figure 6:**
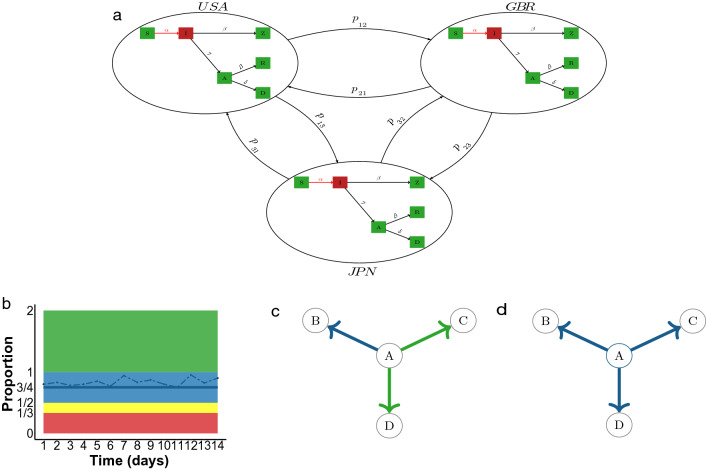
Applying the proposed average control policy in practice: **(a)** schematic of the network meta-population model with travel regulation; **(b)** using the minimum value of the proportion of permissible incoming travel for pairwise zoning of countries; **(c)** travel from Country A to C and from A to D have been assigned the same zone whereas travel from Country A to B has been assigned a different zone; **(d)** simplifying the approach by assigning a country to the most conservative of pairwise zones (here, blue).

Our approach could be implemented similarly to existing policies but using four instead of two zones for more granular control as discussed in Methods. As with existing policies, different quarantine requirements would apply to a traveler based on the zone of the country of departure, and the zone status of each country would be revised every 14 days, as is common with existing policies. As the first step, we estimate model parameters for each country based on the available epidemiological data for the past six months. We then calculate the proportion of daily permissible incoming travel from each country under each policy for the next 14 days. Note that this sequence of proportions of allowed travel from country *i* to country *j*, denoted by $p_{ij}(t)$, fluctuates from day to day. Because daily regulation is impractical, we calculate a summary measure of the sequence of 14 daily proportions of incoming travel. Mean and median would be natural choices, but to be highly conservative, we select the minimum proportion and base our policy for the next 14 days on that minimum. Next, we designate a zone to each country based on this proportion: “red” (0–1/3), “yellow” (1/3–1/2), “blue” (1/2–1), and “green” ($1-\infty$) as in Fig. 6b. Arrivals from a red zone country may be banned completely or passengers may be asked to quarantine for 14 days. Passengers arriving from a yellow or blue zone country are randomly allocated to quarantine on a per-flight basis with the probability of quarantine depending on the zone (yellow vs. blue). The choice of non-quarantine flights must satisfy the constraint that the daily number of flights not exceed the threshold (1/3 for yellow and 1/2 for a blue zone country). Figure 6a demonstrates the proportion of incoming travellers allowed under the proposed policy for a small artificial world of three countries, where $p_{ij}$ is the proportion of people allowed to travel freely from country *i* to country *j* during the next 14 days, $p_{ij} \in \ \{0,1/3,1/2,1\}$ for $i,j = 1,2,3$. The implementation of this approach as described so far is dyadic, i.e., the zone is assigned to a pair of countries, say, from country *i* to country *j*, because it is based on the dyadic proportion $p_{ij}(t)$ (Fig. 6c). To further simplify the approach and align it more closely with existing policies, we can instead calculate a proportion of travel allowed to leave a country, $p_i(t) = \sum _j w_{ij}(t) p_{ij}(t)$, where the weight $w_{ij}(t)$ could be based on travel volume (Fig. 6d). Following a similar approach as above, we can now assign a zone to each country of the world and update these zones every 14 days. If departing passengers are required to provide a negative test result from the past 24 h, as is for example required by the new travel policy to the US^23^ implemented on December 6, 2021, it is possible to relax these regulations further.

Finally, although COVID-19 vaccines are available and vaccination campaigns are underway worldwide, we have a long way to go before the entire world can achieve herd immunity, which has been estimated to be attainable around 2024^37^, if ever^38^. Although vaccines have become readily available in many developed countries, vaccine hesitancy remains high in certain segments of their populations^39–42^. With new variants of the virus emerging in the future, and exiting vaccines being developed against previous strains, countries may need to implement travel restrictions again. For all of these reasons, evidence-based strategies that simultaneously preserve both global public health and the global economy, both in this pandemic and the next, are much needed.

## Methods

### Model

We considered a global travel model where people may travel from one country to another. In this network meta-population model, a node represents a country and an edge represents travel between two countries. The connections between the nodes are modeled using empirical travel data. To model the current state of the pandemic in each country, we used the epidemiological model presented by Warne et al.^29^. In each country, at a given time, the population is divided into six mutually exclusive compartments: susceptible (*S*), undetected infected (*I*), active confirmed (*A*), confirmed recovered (*R*), confirmed deceased (*D*), and unconfirmed removed (*Z*). Undetected infected (*I*) are individuals who have contracted COVID-19 but have not been identified; active confirmed (*A*) are individuals who have been identified as COVID-19 positive but are still receiving treatment or in self-quarantine; recovered confirmed (*R*) are individuals who have been confirmed to have recovered; and confirmed deceased (*D*) are individuals who were reported to have died from COVID-19. Unconfirmed removed (*Z*) are individuals who have recovered or deceased from the disease but who were never confirmed as having contracted the virus. The remaining individuals in the population are susceptible (*S*) and could contract the virus. The spread of disease in each country evolves according to the following transitions and is governed by the indicated parameters as in Fig. 1. Here $\alpha$ is the transmission rate, $\gamma$ is the identification rate, $\beta$ is the recovery rate, and $\delta$ is the death rate.

Using this country-specific model, we then built a global network model of countries utilizing travel data as follows. In the meta-population network model, for a given country *i*, the status of its population is updated in two steps. First, the state of the epidemic evolves based on the internal population of country *i*. The transition from day $t-1$ to *t* is characterized by the shift from $\mathbf {X}_i(t-1)$ based on the local, country-specific epidemiological model, where $\mathbf {X}_i(t-1)$ is a vector of six compartments of the status of country *i* at day $t-1$. Second, the pandemic evolves based on factors external to each country; in this study, the external factor is travelers moving across borders.

### Model parameter estimation

In practice, the epidemiological model parameters for each country are unknown and need to be estimated from empirical data. Most statistical methods rely on the likelihood function for parameter estimation, but because our global model includes unobserved categories (susceptible (*S*), undetected infected (*I*), and unconfirmed recovered (*Z*)), we could not apply either frequentist or Bayesian inferential methods to this problem as both require a tractable likelihood function. Instead, we relied on a class of likelihood-free methods called approximate Bayesian computation (ABC). The use of ABC only requires the ability to forward simulate data from a model given model parameters; the corresponding likelihood function of the model does not need to be evaluated. In this paper, we used a variant method called replenishment ABC (RABC)^43^.

The main challenge of using ABC to calibrate our network meta-population model was the large number of parameters that needed to be estimated. Instead of using ABC to estimate all the parameters for all countries simultaneously, which is computationally expensive and may result in unstable parameter estimates, we used a marginal estimation strategy to estimate each country’s epidemiological parameters separately, while still taking the travel data into account. For a given country *i*, we first reconstructed all six states describing the pandemic situation in all other countries $j \ne i$ based on their epidemiological data. Based on the travel data, we then estimated the number of cases imported to country *i* from other countries. These quantities, together with the epidemiological data for country *i*, were then used to estimate the parameters for country *i*. More details on the estimation procedure are available in the Supplementary Materials.

### Traffic regulation policies

Leaving borders completely open puts a country’s public health at risk, while closing borders is likely to have a negative effect on the economy. A policy that finds a middle ground between these two extremes is expected to provide a better balance between maintaining public health and preserving the economy. Some commonly used policies to ease travel restrictions include a 14-day quarantine for people traveling from high-risk regions and a 14-day quarantine requirement for all arrivals. However, there are no theoretical results demonstrating that these approaches control the pandemic as well as a full border closure would.

Under the 14-day quarantine for all arrivals policy, undetected infectious individuals transition to either active confirmed, confirmed recovered, confirmed deceased, or unconfirmed removed as a result of monitoring during the quarantine period. As such, this policy helps stop importation of new undetected cases. However, this approach is also likely to dissuade travelers. Furthermore, if a large number of people are willing to travel despite the quarantine requirement, the country may see a surge in active confirmed cases from individuals undergoing mandatory quarantine. This surge could strain the receiving country’s healthcare system. To encourage travel, some countries have relaxed the quarantine requirement by dividing other countries into zones based on risk: travelers arriving from high-risk countries need to quarantine for 14 days whereas those arriving from low-risk countries have no quarantine requirement. While this approach could revitalize travel, it may still risk overburdening the receiving country’s healthcare system. Therefore, policy is needed that avoids these drawbacks and offers some guarantees that the pandemic remains under control.

In our global travel model, at the end of each day, we updated travel according to

$$\begin{aligned} \mathbf {X}_i^+(t) = \mathbf {X}_i(t) + \sum \limits _{1 \le j \ne i \le n} \mathbf {f}_{ji}^{\text {out}}(t) - \mathbf {f}_i^{\text {out}}(t), \end{aligned}$$

where $\mathbf {f}_{ji}^{\text {out}}(t)$ denotes the six compartments of individuals traveling from country *j* to country *i*. Our goal was to regulate the volume of inbound travel by only letting a certain proportion of travelers enter a country each day. We denote the proportions sequence $0 \le \{ p_{ji}(t) \}_{1 \le j \ne i \le n } \le 1$ as the travel regulation sequence from country *j* to country *i* at day *t*, i.e., a temporal sequence of proportions of travelers permitted. A total shutdown of inbound travel in country *i* at day *t* is equivalent to $p_{ji}(t) = 0, \forall \, 1 \le j \ne i \le n$, and fully open inbound travel in country *i* at day *t* is equivalent to $p_{ji}(t) = 1, \forall \, 1 \le j \ne i \le n$. Under our strategy, at the end of day *t*, the status of country *i* is updated as follows:

$$\begin{aligned} \mathbf {X}_i^+(t) = \mathbf {X}_i(t) + \sum \limits _{1 \le j \ne i \le n} p_{ji}(t) \mathbf {f}_{ji}^{\text {out}}(t) - \mathbf {f}_i^{\text {out}}(t). \end{aligned}$$

As a result, the number of undetected infected cases in country *i* at day *t* is also updated as $I_i^+(t) = I_i(t) + \sum \limits _{1 \le j \ne i \le n} p_{ji}(t) I_{ji}^{\text {out}}(t) - I_i^{\text {out}}(t)$. In our model, the undetected infected category is the only one that directly drives the epidemic. Therefore, if we can find a sequence $\{ p_{ji}(t) \}_{1 \le j \ne i \le n }$ that ensures the number of undetected cases during the regulation period *T* does not go above a desired threshold *c*, then this sequence could be used to regulate travel. We took the following approach to find such a sequence. Consider a specific country with *I*(0) undetected cases initially, and suppose that under our regulation policy, we allow the number of daily undetected infected to be inflated at a rate *p*. In other words, if *I*(*t*) is the number of undetected cases evolved from the internal pandemic in a country at day *t*, then we allow incoming travel such that the number of undetected cases can increase up to $I^+(t) = I(t)(1 + p)$. Our goal is to find the value *p* so that the number of daily undetected infected cases during the regulation period stays below a given threshold *c*. Based on this value and the pandemic situation in the departure country, we can determine an appropriate sequence of proportions.

We considered two types of regulation. Regulation in terms of *average control* entails finding a proportion *p* such that the average number of daily undetected cases in the next *T* days stays below a fixed threshold *c*. Regulation in terms of *probability control* entails finding a proportion *p* such that the probability of daily undetected cases in the next *T* days staying lower than a threshold *c* is at least $\pi$. Lemma 1 and Lemma 2 in the Supplementary Materials allowed us to find the proportion *p*.

Figure 3a shows the number of undetected infected cases in a country in the 7 days following the implementation of three different policies: fully open, fully closed, and our proposed average control policy with a threshold of $c = 70$. Note that the number of undetected cases under the average control scenario is below the required threshold and does not differ much from the one obtained under the fully closed scenario. Additionally, based on our calculations, the volume of inbound travelers under the average control policy can be up to $88.64\%$ of the normal load. For more mathematical details on these calculations, including the proof, see the Supplementary Materials.

In practice, it may be hard to apply the proposed average control policies due to the logistical difficulties in regulation travel proportions daily. Therefore, we simplified these policies by first calculating the minimum value of the proportion sequence of incoming travelers. We then assigned the proportion of incoming travelers allowed as 0, 1/3, 1/2,  or 1 if this minimum value belongs to ranges [0, 1/3), [1/3, 1/2), [1/2, 1), or $[1, \infty )$, respectively.

### Evaluation of travel regulation effectiveness

Travel regulation effectiveness was evaluated based on two factors: the percentage of inbound travel and the epidemiological situation in the target country. We evaluated inbound travel in two ways. The percentage of inbound travel capacity is the number of inbound travelers allowed under the policy divided by the number of inbound travelers under normal circumstances. The expected percentage of inbound travel is an adjusted version of the percentage of inbound travel capacity; if the 14-day quarantine policy is applied to people departing a country, we assume that only 5$\%$ of travelers from this country are willing to travel. South Korea requires a 14-day quarantine for all arrivals, and data provided by the Korea Tourism Organization supports this 5$\%$ assumption^24^. After this adjustment, the percentage of expected inbound travelers is obtained by dividing the number of expected inbound travelers by the number of inbound travelers under normal circumstances, which gives us insight into the effect of the 14-day quarantine requirement. We report the effectiveness of policies on the epidemiological situation in the receiving country using three factors: the relative change in cases, the relative change in confirmed cases, and the percent of travelers who will eventually move to the active confirmed category after arrival. Relative change in cases is the difference between number of cases (detected and undetected) at the end and at the beginning of the regulated period divided by the number of cases at the beginning of the period; similarly, relative change in confirmed cases is the difference in the number of detected cases at the end and at the beginning of the regulated period divided by the number of detected cases at the beginning of the period. The percent of travelers who will eventually move to the active confirmed category after arrival is calculated by using the number of undetected travelers who were eventually confirmed as having COVID-19 divided by the total number of incoming travelers.

### Real data analysis

To identify the change point, we first identify zero crossings of the second derivative of the cumulative incidence function, i.e., where the function changes from convex to concave, and then use $\alpha _1$ for the pre-period and $\alpha _2$ for the post-period. To reduce noise, we use a 7-day moving average, rather than daily values, to detect these change points. The initial condition for each country is chosen as (*S*(0), *I*(0), *A*(0), *R*(0), *D*(0), *Z*(0)), where *A*(0), *R*(0), *D*(0) are obtained from the real data, and assign $Z(0) = 0$. We obtain *I*(0) by simulating each country independently as in Warne et al.^29^, where we allow *I*(0) to follow a uniform distribution with a range from 0 to 50 ⋅ *U*(0), where *U*(0) is the total confirmed cases in the country on that day. The median value of the posterior for *I*(0) is used as a point estimate for *I*(0). Due to reporting delays and data quality concerns, especially for the recovered confirmed cases, we only used the daily confirmed cases and daily deceased to construct the distance when performing the ABC algorithm to study the model’s parameters. The distance chosen for the calibration was the standardized Euclidean distance

$$\begin{aligned} \text{ Distance }= \frac{1}{T} \left[ \sqrt{ \sum _{t=1}^T \frac{(U(t)-U^{(s)}(t))^2}{\sigma _U(t)}}+ \sqrt{ \sum _{t=1}^T \frac{(D(t)-D^{(s)}(t))^2}{\sigma _D(t)}}\right] , \end{aligned}$$

where $t=1,\ldots ,T$ are the days during the study period, *U*(*t*) is the total daily confirmed cases, *D*(*t*) is the daily deceased cases in that country at day *t*, and $U^{(s)}(t)$ and $D^{(s)}(t)$ are the daily cases from simulated data. The prior standard deviations $\sigma _U(t)$ and $\sigma _D(t)$ were obtained by simulating the data for different combinations of parameters during the study period and keeping 1000 realizations with the total number of confirmed and deceased cases at the end of the period no more than 5$\%$ different compared to the real data. To avoid the high rejection rate, we added one step by first running a preliminary analysis on each country independently and obtaining the posterior distribution for the parameters. We then used these parameters to simulate the data. To encourage more diversity in the realizations, we replaced the parameter $\alpha$ with $\alpha + K_\alpha$, where $K_\alpha$ is a random number drawn from a uniform distribution from $-\alpha /2$ to $\alpha /2$. Based on these 1000 realizations, we then calculated $\sigma _D(t)$ and $\sigma _U(t)$ for $t =1,\ldots ,T$.

## Data and materials availability

Proprietary flight data for January and February of 2020 are commercially available from the Official Airline Guide (OAG). The flight tracking data is publicly available from the OpenSky Network database at https://doi.org/10.5281/zenodo.4670229. The COVID-19 data is publicly available from the Johns Hopkins University coronavirus data repository https://github.com/CSSEGISandData/COVID-19. Reformatted data and R code used in this study are publicly available at https://github.com/onnela-lab/covid-travel.

## Supplementary Information

Supplementary Information.
